# Absence of p.R50X *Pygm* read-through in McArdle disease cellular models

**DOI:** 10.1242/dmm.043281

**Published:** 2020-01-13

**Authors:** Guillermo Tarrasó, Alberto Real-Martinez, Marta Parés, Lídia Romero-Cortadellas, Laura Puigros, Laura Moya, Noemí de Luna, Astrid Brull, Miguel Angel Martín, Joaquin Arenas, Alejandro Lucia, Antoni L. Andreu, Jordi Barquinero, John Vissing, Thomas O. Krag, Tomàs Pinós

**Affiliations:** 1 Mitochondrial and Neuromuscular Disorders Unit, Vall d'Hebron Institut de Recerca, Universitat Autònoma de Barcelona, Barcelona 08035, Spain; 2 Centro de Investigación Biomédica en Red de Enfermedades Raras (CIBERER), Madrid 28029, Spain; 3 Gene and Cell Therapy Unit, Vall d'Hebron Institut de Recerca, Universitat Autònoma de Barcelona, Barcelona 08035, Spain; 4 Laboratori de Malalties Neuromusculars, Institut de Recerca Hospital de la Santa Creu i Sant Pau, Universitat Autònoma de Barcelona, Barcelona 08041, Spain; 5 Sorbonne Université, INSERM UMRS_974, Center of Research in Myology, 75013 Paris, France; 6 Mitochondrial and Neuromuscular Diseases Laboratory, 12 de Octubre Hospital Research Institute (i+12), Madrid 28041, Spain; 7 Faculty of Sport Sciences, European University, Madrid 28670, Spain; 8 Copenhagen Neuromuscular Center, Department of Neurology, Rigshospitalet, University of Copenhagen, Copenhagen DK-2100, Denmark

**Keywords:** McArdle disease, Metabolic myopathy, Premature termination codon, Read-through, Cellular models

## Abstract

McArdle disease is an autosomal recessive disorder caused by the absence of muscle glycogen phosphorylase, which leads to blocked muscle glycogen breakdown. We used three different cellular models to evaluate the efficiency of different read-through agents (including amlexanox, Ataluren, RTC13 and G418) in McArdle disease. The first model consisted of HeLa cells transfected with two different GFP-*PYGM* constructs presenting the *Pygm* p.R50X mutation (GFP-*PYGM* p.R50X and *PYGM* Ex1-GFP p.R50X). The second cellular model was based on the creation of HEK293T cell lines stably expressing the *PYGM* Ex1-GFP p.R50X construct. As these plasmids encode murine *Pygm* cDNA without any intron sequence, their transfection in cells would allow for analysis of the efficacy of read-through agents with no concomitant nonsense-mediated decay interference. The third model consisted of skeletal muscle cultures derived from the McArdle mouse model (knock-in for the p.R50X mutation in the *Pygm* gene). We found no evidence of read-through at detectable levels in any of the models evaluated. We performed a literature search and compared the premature termination codon context sequences with reported positive and negative read-through induction, identifying a potential role for nucleotide positions −9, −8, −3, −2, +13 and +14 (the first nucleotide of the stop codon is assigned as +1). The *Pygm* p.R50X mutation presents TGA as a stop codon, G nucleotides at positions −1 and −9, and a C nucleotide at −3, which potentially generate a good context for read-through induction, counteracted by the presence of C at −2 and its absence at +4.

## Abstract (precis)

**Summary:** Here, we evaluated the efficiency of different read-through agents in McArdle disease cell culture models, revealing that read-through compounds do not restore full-length muscle glycogen phosphorylase.

## INTRODUCTION

McArdle disease (glycogen storage disease type V, OMIM^®^ 232600) is an autosomal recessive disorder of glycogen metabolism that was first described in 1951 (Mc, 1951). This condition is one of the most frequent metabolic myopathies, with an estimated prevalence in the Caucasian population of 1/139,000 and both sexes similarly affected (Santalla et al., 2017). McArdle disease is caused by pathogenic mutations in both copies of the gene (*PYGM*) encoding the muscle isoform of glycogen phosphorylase (GP-M), generally leading to loss of activity and inability to break down glycogen, making this source of energy unavailable to patients (Mc, 1951; Tsujino et al., 1995; Quinlivan et al., 2010; Lucia et al., 2012; Bartram et al., 1993; Dimauro et al., 2002). Patients present with exercise intolerance, usually in the form of reversible, acute crises of early exertional fatigue and contractures that can also be accompanied by rhabdomyolysis and myoglobinuria (‘dark urine’) (Santalla et al., 2017; Lucia et al., 2012). Although more than 150 pathogenic *PYGM* mutations have been reported since the first pathogenic variants were described in 1993 (Bartram et al., 1993; Nogales-Gadea et al., 2015; Tsujino et al., 1993), clearly the most prevalent among Caucasian patients is the nonsense p.R50X (c.148C>T) mutation, with an allele frequency of ∼50% (Santalla et al., 2017; Quinlivan et al., 2010; Lucia et al., 2012; Bruno et al., 2006; Bartram et al., 1994; el-Schahawi et al., 1996; Martin et al., 2004; Aquaron et al., 2007; Gurgel-Giannetti et al., 2013; Deschauer et al., 2007). Nonsense mutations like the p.R50X variant introduce premature termination codons (PTCs) into the protein-coding gene sequence, thereby leading to premature termination of translation and, as a consequence, to the production of truncated proteins (Mendell and Dietz, 2001). PTCs are often associated with severe disease phenotypes [and account for ∼12% of all described genetic alterations causing human inherited conditions (Mort et al., 2008)]. In McArdle disease, ∼35% of all mutations other than p.R50X generate PTCs (Aquaron et al., 2007; Deschauer et al., 2007; Rubio et al., 2006, 2007; Nogales-Gadea et al., 2007). Furthermore, ∼75% of all patients (251 of 333) in the Spanish registry of McArdle disease present a PTC in at least one of the two gene copies (Santalla et al., 2017).

There is currently no curative therapy for McArdle disease. Gene therapy has been evaluated in the spontaneously occurring McArdle sheep model and recently in the knock-in (p.R50X homozygous) McArdle mouse model (McNamara et al., 2019; Howell et al., 2008). In the sheep model, intramuscular application of two different vectors (adenovirus 5 vector and an adeno-associated virus serotype 2) containing GP-M expression cassettes produced only local expression of functional GP-M, and the number of GP-M-expressing fibers decreased with time (Howell et al., 2008). Furthermore, intraperitoneal injection of recombinant adeno-associated virus serotype 8 containing a functional copy of *Pygm* in the McArdle mouse model resulted in *Pygm* expression, improved skeletal muscle architecture, reduced accumulation of glycogen and restoration of voluntary running wheel activity, but a lack of improvement in the hanging wire ability (McNamara et al., 2019). Thus, further studies are required in this field before this approach can be proposed in patients. Several drug treatments have also been evaluated *in vitro* or *in vivo*. Valproate, an inhibitor of histone deacetylation, was shown to induce a remarkable decrease in glycogen levels by increasing the expression of the brain isoform of glycogen phosphorylase in skeletal muscle cultures derived from the knock-in (p.R50X homozygous) McArdle mouse model (de Luna et al., 2015). Similarly, treatment of the McArdle sheep model with valproate induced the re-expression of the brain/developmental glycogen phosphorylase in their muscle fibers, although this effect was not accompanied by an increase in exercise capacity (Howell et al., 2015). However, the fact that PTC-causing mutations are the most prevalent pathogenic variants in McArdle disease patients provides biological rationale to assess the effectiveness of the treatment with read-through agents (RTAs) such as aminoglycosides, which are known to induce the ribosome to bypass a PTC (Bidou et al., 2012). In this regard, a preliminary trial with short-term (10-day) gentamicin treatment in McArdle disease patients with a PTC failed to normalize ^31^P-magnetic resonance spectroscopy indicators of GP-M deficiency in muscle (Schroers et al., 2006). However, read-through of the *PYGM* p.R50X mutation was reported in non-muscle culture cell models (Chinese hamster ovary cells) that had been transiently transfected with p.R50X-GFP plasmid constructs and treated with the aminoglycoside G418 (Birch et al., 2013). Other low-molecular RTAs were reported to induce ‘read-through’ in PTCs, including 3-[5-(2-fluorophenyl)-1,2,4-oxadiazol-3-yl]-benzoic acid (also known as PTC124, Ataluren or Translarna) (Welch et al., 2007), RTC13 and RTC14 (Du et al., 2009a) and amlexanox (Gonzalez-Hilarion et al., 2012), among others. Thus, it was the purpose of our study to assess the efficacy of different RTAs in (1) transiently transfected cells with p.R50X plasmid constructs, (2) cells stably expressing these constructs and (3) primary skeletal muscle cells derived from the McArdle mouse model.

## RESULTS

### Absence of read-through effect on transiently transfected HeLa cells

**Fig. 1.**
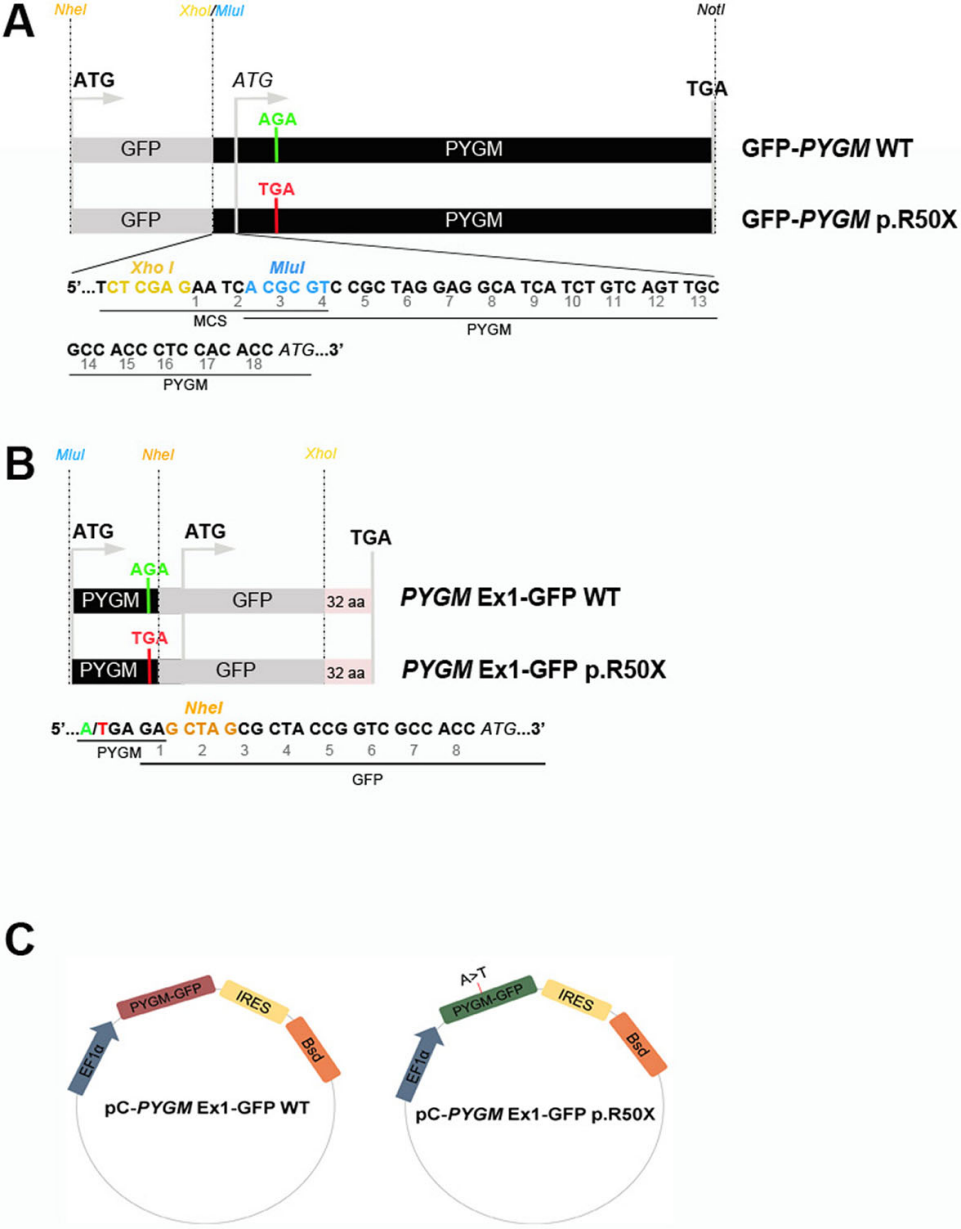
**GFP-*PYGM* plasmid constructs used to analyze p.R50X *Pygm* read-through.** (A-C) Different GFP-*PYGM* constructs were transiently (A,B) and stably (C) transfected in HeLa and HEK293T cells, respectively. (A) The GFP-*PYGM* wild-type (WT) and p.R50X constructs included the GFP encoding sequence followed by the full-length mouse *Pygm* cDNA encoding sequence, and varied in the absence or presence of the p.R50X mutation. The *XhoI* restriction site (yellow) disrupted the GFP coding sequence corresponding to the last 20 amino acids of the GFP protein. The *MluI* (blue) restriction site was used to insert the *Pygm* cDNA into the pCI-neo multicloning site. (B) The *PYGM* Ex1-GFP WT and p.R50X constructs consisted of the mouse exon 1 *Pygm* cDNA encoding the first 50 amino acids of the GP-M protein followed by the GFP encoding sequence. *MluI* (blue) and *NheI* (orange) restriction sites were used to insert the *Pygm* sequence, while *NheI* and *XhoI* (yellow) restrictions sites were used to insert the GFP encoding sequence into the pCI-neo multicloning site. (C) Structure of pC-*PYGM* EX1-GFP WT (left) and pC-*PYGM*-EX1-GFP p.R50X (right). EF1α, elongation factor 1α; GFP, enhanced green fluorescent protein; IRES, internal ribosomal entry site; Bsd, blasticidin S resistance gene.

**Fig. 2.**
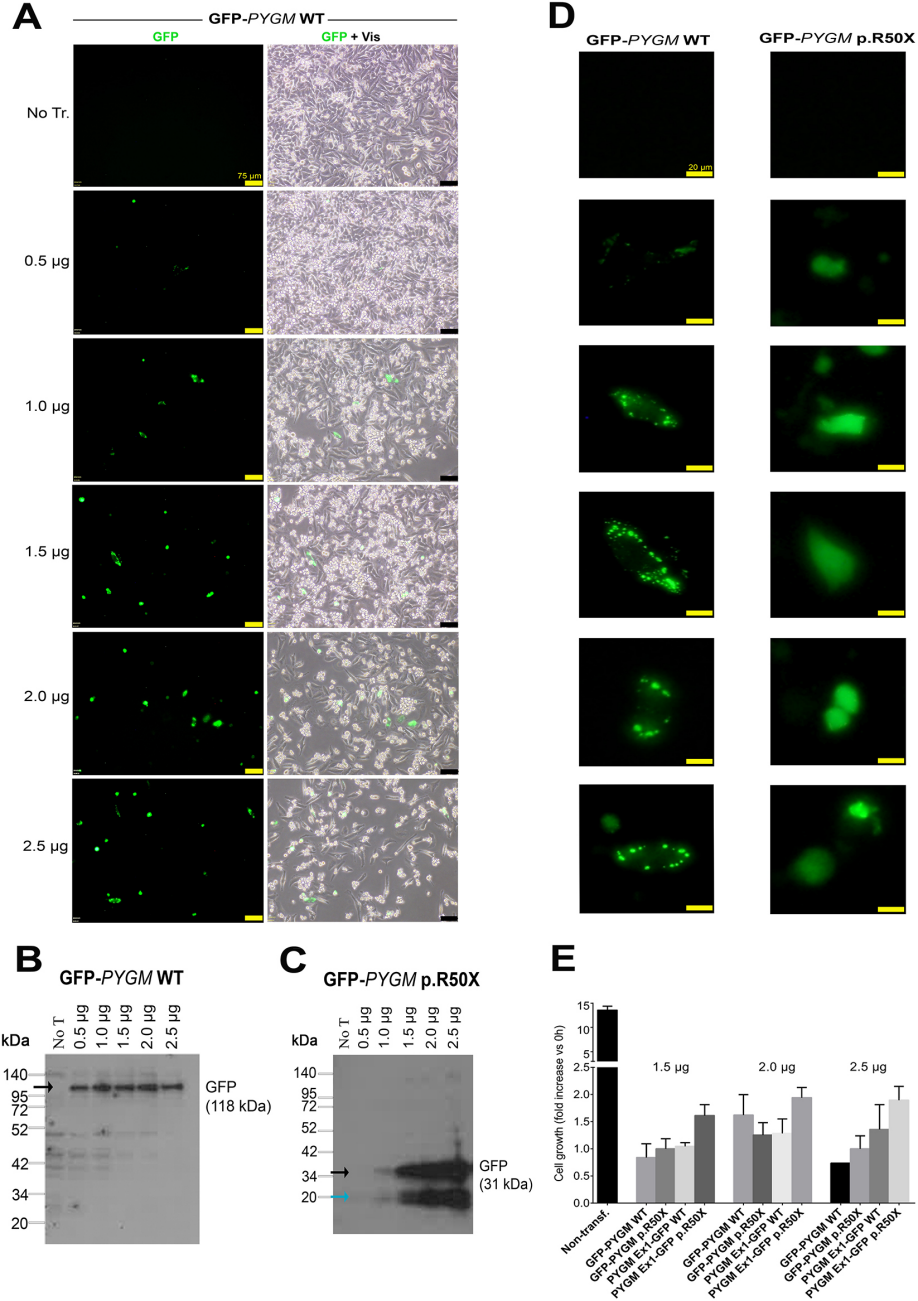
**Transfection efficiency analysis using different amounts of the GFP-*PYGM* plasmids.** (A) GFP *PYGM* fluorescence analysis of HeLa cells transfected with different amounts of GFP-*PYGM* WT. Images were obtained 24 h after transfection. Scale bars: 75 μm. (B,C) Western blot analyses of HeLa cells transfected with different amounts of GFP-*PYGM* WT and p.R50X plasmids. In both B and C, measures were performed 72 h after transfection. Black arrows mark specific protein bands; the blue arrow marks unspecific protein band. (D) GFP-*PYGM* fluorescence details of cells transfected with GFP-*PYGM* WT and p.R50X plasmids. Images were obtained 24 h after transfection. Scale bars: 20 μm. (E) Cell growth studies of HeLa cells transfected with 1.5, 2 and 2.5 µg GFP-*PYGM* WT and p.R50X plasmids. Two independent experiments were performed in each condition. Error bars indicate s.d. between the two independent experiments.

**Fig. 3.**
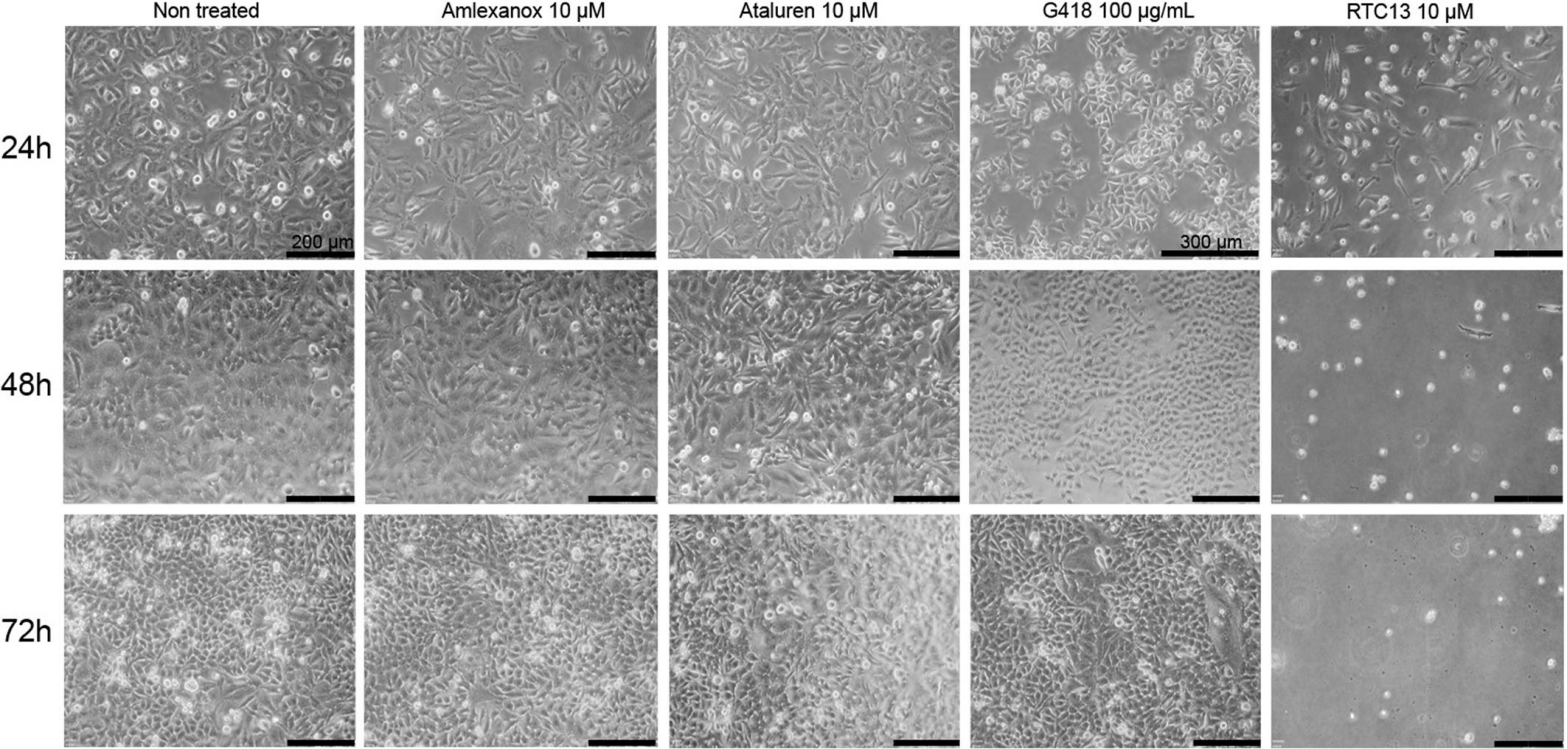
**Viability of HeLa cells after treatment with amlexanox, Ataluren, G418 or RTC13.** Results were obtained after 24, 48 and 72 h of treatment with amlexanox (10 µM), Ataluren (10 µM), G418 (100 µg/ml) and RTC13 (10 µM). Scale bars: 200 µm unless indicated.

**Fig. 4.**
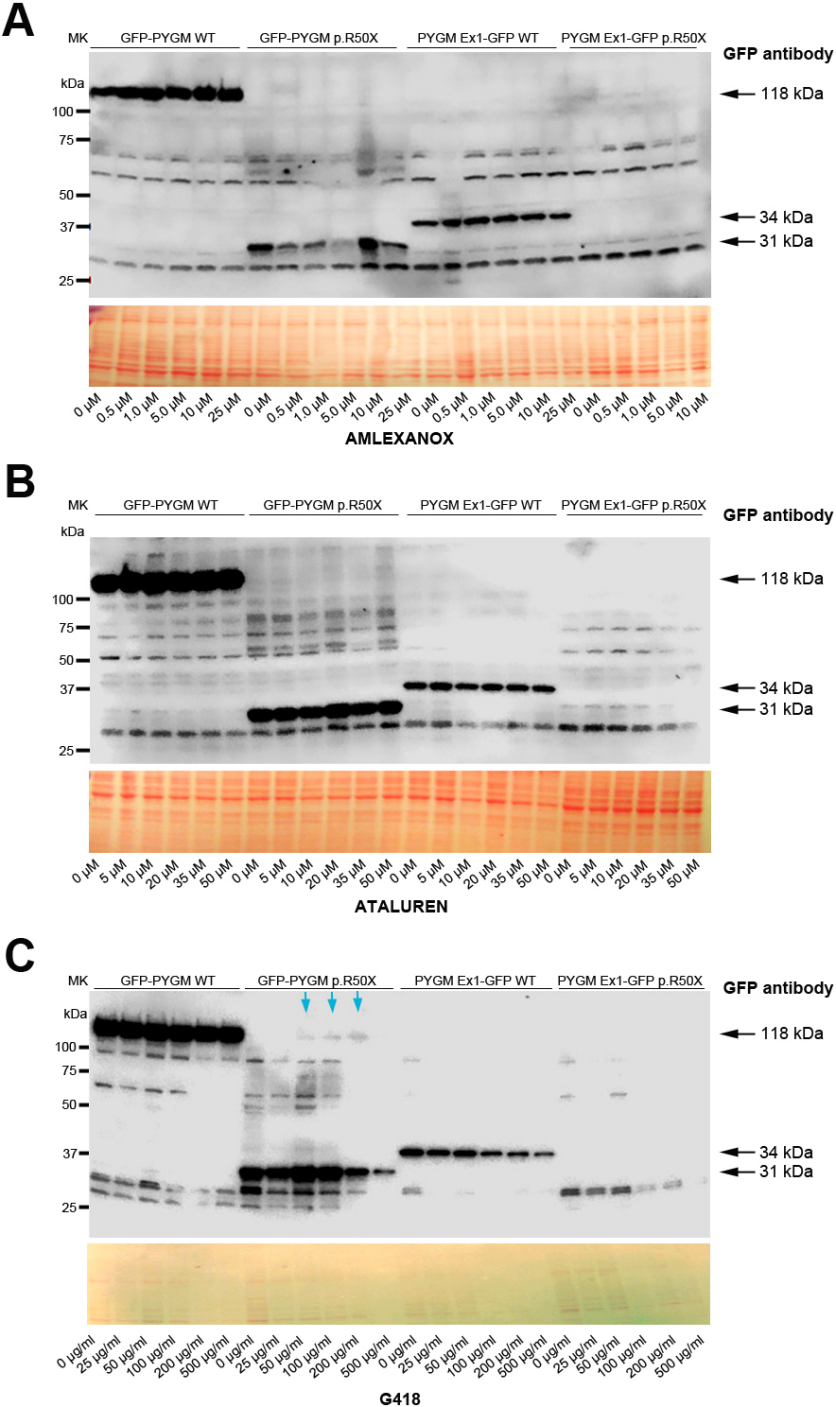
**Read-through analysis in transient transfected cells.** (A-C) Read-through analysis in HeLa cells transfected with WT, p.R50X GFP-PYGM and PYGM-GFP plasmids after 72 h treatment with amlexanox (A), Ataluren (B) and G418 (C). The GFP-*PYGM* WT plasmid generates a fusion protein of 1079 amino acids and a molecular weight of 118 kDa, consisting of the N-terminal GFP protein (219 amino acids) fused to the full-length *PYGM* protein (18 amino acids from the 5′UTR+842-amino-acid full-length protein). In the absence of read-through induction, the GFP-*PYGM* p.R50X plasmid generates a fusion protein of 286 amino acids (GFP 219 amino acids+18 amino acids *PYGM* 5′UTR and 49 amino acids from *PYGM* coding sequence) and a molecular weight of 31 kDa. The *PYGM*-Ex1 GFP WT plasmid generates a fusion protein of 309 amino acids and molecular weight of 34 kDa, consisting of the first 50 amino acids of the GP-M protein fused to the 219 amino acids from the GFP coding sequence and the 40 unspecific extra amino acids (8 and 32 in the N- and C-terminus of GFP protein, respectively) derived from the cloning process. Finally, in the absence of read-through induction, the *PYGM*-Ex1 GFP p.R50X plasmid generates a protein consisting only of the first 49 amino acids (∼5 kDa) of the GP-M protein. Blue arrows in C indicate potential read-through obtained for the GFP-*PYGM* p.R50X construct after treatment with G418, which could not be replicated in further experiments.

We first evaluated the capacity of the different compounds to induce read-through of the p.R50X mutation in the *Pygm* gene in cells with high levels of *Pygm* expression, in conditions in which the *Pygm* mRNA is not subjected to the nonsense-mediated decay (NMD) process, a specialized mRNA surveillance mechanism that aims to reduce the synthesis of deleterious C-terminally truncated proteins in eukaryotic organisms (Kayali et al., 2012). We used HeLa cells transfected with four different plasmids that contained the wild-type (WT) or mutated (p.R50X) *Pygm* complementary DNA (cDNA) sequence (GFP-*PYGM* WT and p.R50X, and *PYGM* Ex1-GFP-WT and p.R50X) (Fig. 1A,B). In order to optimize transfection efficiency, different amounts of the GFP-*PYGM* WT plasmid were transfected in HeLa cells and GFP fluorescence was analyzed 24 h after transfection. There was a positive correlation between the amount of GFP-*PYGM* fluorescence signal and the quantity of GFP-*PYGM* WT plasmid transfected in these cells (Fig. 2A). Additionally, a moderate decrease in cell confluency was observed in HeLa cells transfected with 2.0 µg and 2.5 µg of GFP-*PYGM* WT plasmid compared to cells transfected with 0.5, 1.0 and 1.5 µg, indicating a reduction in cell viability with higher amounts of plasmid transfection (Fig. 2A). When western blot analyses were performed, similar amounts of GFP-*PYGM* protein were observed in cells transfected with 0.5, 1, 1.5, 2 and 2.5 µg of GFP-*PYGM* WT plasmid (Fig. 2B). However, a positive correlation was observed between GFP-*PYGM*_p.R50X_ band intensity and the amount of transfected plasmid (Fig. 2C). Interestingly, HeLa cells transfected with GFP-*PYGM* WT plasmid presented a cytosolic punctuate GFP-*PYGM* localization, whereas those transfected with the GFP-*PYGM* p.R50X plasmid showed a diffuse GFP-*PYGM*_p.R50X_ localization (Fig. 2D). Overall, these results suggest that higher levels of WT and GFP-*PYGM*_p.R50X_ protein are obtained when 1.5, 2.0 and 2.5 µg of both plasmids are transfected, although they might imply a reduction in cell viability. In this regard, cell growth was analyzed in HeLa cells 72 h after transfection with 1.5, 2.0 and 2.5 µg of the distinct plasmids, and, although no major differences were observed, a trend for higher cell growth in cells transfected with 2.0 µg of plasmids was observed (Fig. 2E). Thus, as a compromise, 2 µg of plasmid was used in the subsequent experiments. Next, we evaluated the potential toxicity of the different RTAs (amlexanox, Ataluren, RTC13 and G418) in HeLa cells. Thus, cells were treated for 72 h with either amlexanox, Ataluren, RTC13 or G418 at doses that have previously been shown to induce read-through in different cell cultures with no apparent toxicity [i.e. 10 µM for amlexanox (Gonzalez-Hilarion et al., 2012), PTC124 (Lentini et al., 2014; Du et al., 2013; Kayali et al., 2012) or RTC13 (Du et al., 2013; Kayali et al., 2012; Nakamura et al., 2012), and 100 µg/ml for G418 (Howard et al., 1996; Heier and DiDonato, 2009; Friesen et al., 2018; Floquet et al., 2011)]. No significant changes in cell growth were observed in HeLa cells treated with amlexanox, Ataluren or G418 compared to non-treated cells (Fig. 3). However, a progressive reduction in cell number was observed between 24 h and 72 h in HeLa cells treated with RTC13 (Fig. 3). Consequently, RTC13 was not further evaluated as a potential RTA in this cell model. Finally, when the read-through capacity for amlexanox, Ataluren and G418 was tested in transfected cells, no read-through effect was observed in amlexanox- or Ataluren-treated cells, as the 118 kDa and 34 kDa bands corresponding to the full-length translation of the GFP-*PYGM* p.R50X and *PYGM* Ex1-GFP p.R50X were not detected (Fig. 4A,B). However, a faint 118 kDa band was observed in GFP-PYGM p.R50X-transfected cells when treated with 50, 100 and 200 mg/ml G418 (Fig. 4C). An attempt to reproduce this specific result failed, thus a read-through effect caused by the G418 treatment could not be confirmed.

### Absence of read-through effect on stably transfected HEK293T cells

**Fig. 5.**
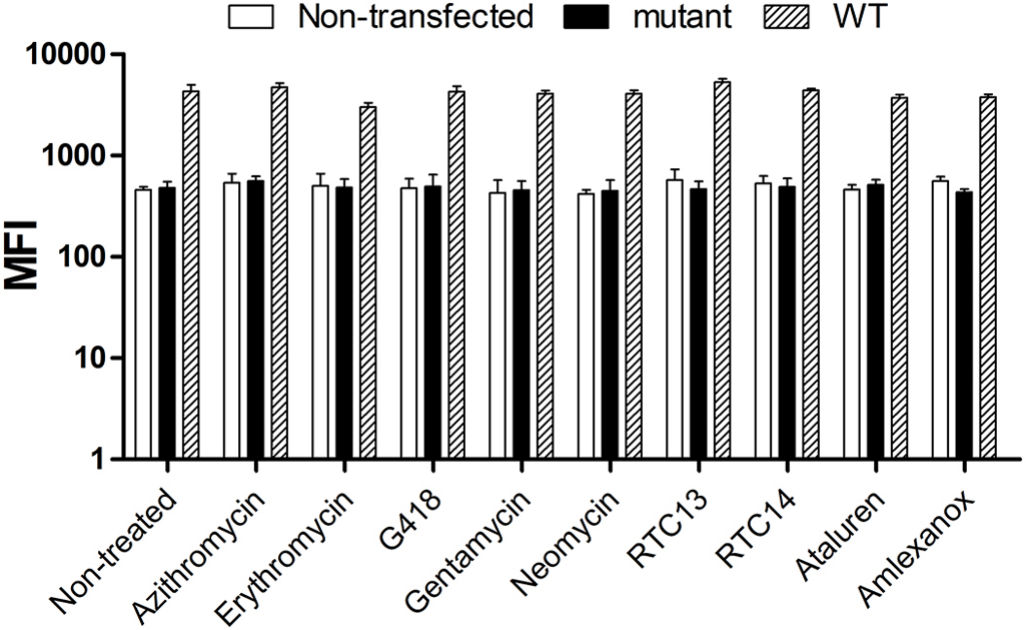
**Flow-cytometric analysis of the read-through effect 48 h after treatment.** Mean fluorescence intensity (MFI) in non-transfected cells and in those stably expressing the WT and the p.R50X constructs. Azithromycin and geneticin, 50 μg/ml; erythromycin, gentamycin and neomycin, 100 μg/ml; RTC13 and RTC14, 10 μM; Ataluren and amlexanox, 30 μM. Experiments were performed in duplicate for all conditions. Error bars indicate s.d. from duplicate experiments.

A wider panel of RTAs was used to induce read-through in human embryonic kidney 293T (HEK293T) cells stably expressing the WT and the pC-*PYGM* Ex1-GFP WT and p.R50X constructs (Table 1). In a set of preliminary experiments, we tested different ranges of drug concentrations and measured cell viability by flow cytometry after labeling with 7-aminoactinomycin (7-AAD). Based on these results, we established the optimal dose of the drugs and determined that the optimal time for analysis was 48 h after drug exposure (data not shown). None of the RTAs tested increased the mean fluorescence intensity (MFI) (Fig. 5) or the percentages of GFP^+^ cells (data not shown) in cells carrying the mutated form of *Pygm*, indicating the lack of read-through effect.

**Table 1.**
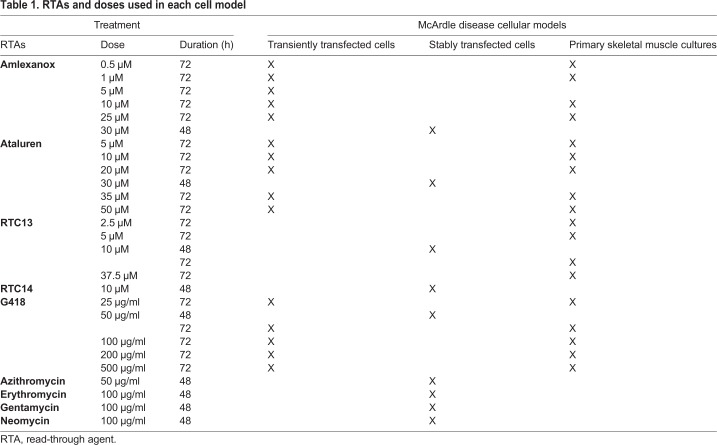
**RTAs and doses used in each cell model**

### Primary cultures treated with read-through compounds present NMD

**Fig. 6.**
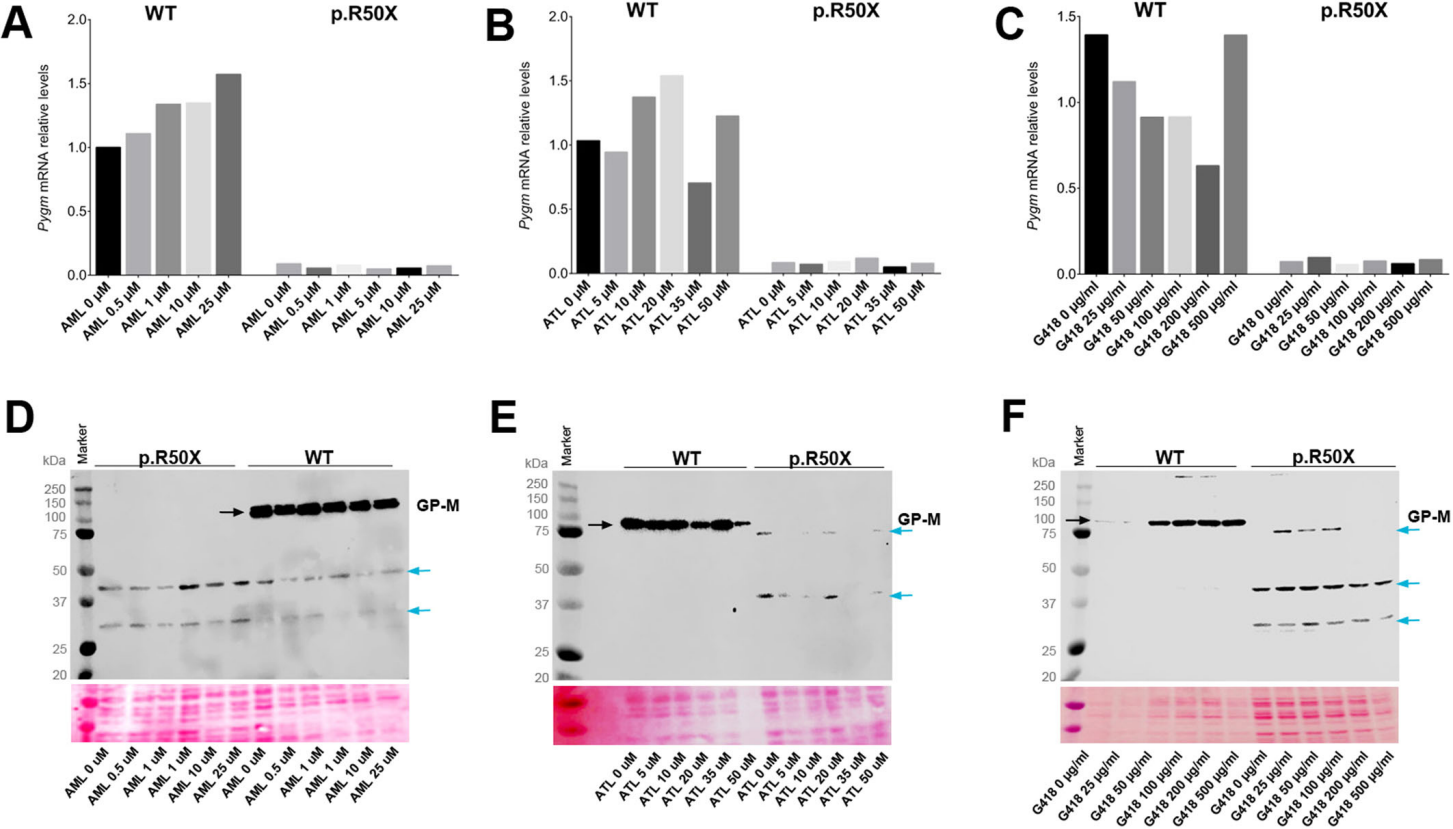
**Nonsense-mediated decay (NMD) and read-through analysis in McArdle myotubes.** (A-C) *PYGM* mRNA levels in WT and McArdle mouse myotubes after 72 h treatment with different concentrations of amlexanox (AML) (A), Ataluren (ATL) (B) and G418 (C), respectively. (D-F) GP-M protein levels in WT and McArdle mouse myotubes after 72 h treatment with different concentrations of AML (D), ATL (E) and G418 (F), respectively. Black arrows mark the specific PYGM protein band (94 kDa); blue arrows mark unspecific protein bands.

We next evaluated whether amlexanox, Ataluren and G418 were able to suppress the NMD of *Pygm* mRNA in the skeletal muscle cultures derived from the McArdle mouse model. In this case, no significant increase in *Pygm* mRNA levels was observed for any of the drugs and doses tested (Fig. 6A-C). Therefore, these results indicate that amlexanox, Ataluren and G418 are unable to suppress NMD in this model.

### Absence of read-through effect on primary cultures

In western blot analyses, the 94 kDa band corresponding to the full-length GP-M protein was observed in treated and untreated WT myotubes (Fig. 6D-F; Fig. S1). By contrast, no specific bands were detected in McArdle mouse myotubes for any dose of the four compounds tested (amlexanox, Ataluren, RTC13 and G418) (Fig. 6D-F; Fig. S1). The absence of full-length *PYGM* protein in treated McArdle myotubes suggests the lack of read-through effect. These findings are consistent with the results from transiently and stably transfected cells.

### Analysis of mouse *Pygm* p.R50X nucleotide context sequence

**Fig. 7.**
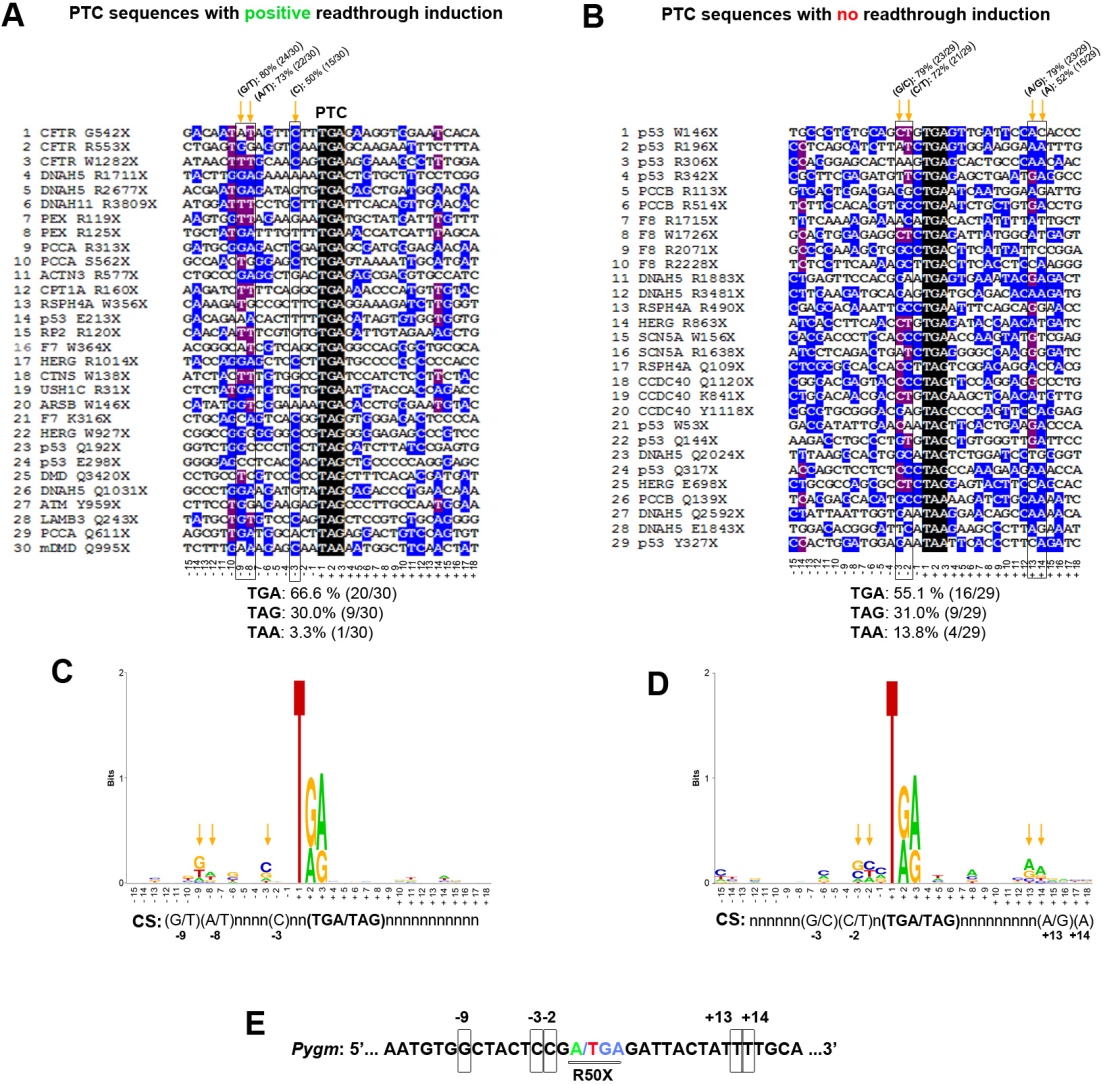
**Analysis of PTC sequences with positive and negative read-through induction.** (A) Multiple Alignment using Fast Fourier Transform (MAFFT) of 30 sequences with described positive read-through induction. (B) MAFFT alignment of 29 sequences with reported absence read-through induction. Orange arrows mark highly conserved nucleotides (>70% for the combination of two nucleotides and ≥50% for a single nucleotide) among the aligned sequences. (C) Consensus sequence obtained from the 30 sequences with described read-through induction. (D) Consensus sequence obtained from the 29 sequences with reported absence of read-through induction. (E) Mouse *Pygm* PTC context sequence. Potentially relevant nucleotides for read-through induction are squared.

As the read-through efficiency is influenced by the stop codon composition and its surrounding sequence (Howard et al., 2004), we wanted to determine whether the absence of read-through induction could be caused by the nature of the *Pygm* p.R50X PTC and its surrounding sequence. To this end, we needed to establish which nucleotide/s surrounding the PTC might influence the read-through induction caused by the different compounds. Thus, we analyzed the context sequence [15 nucleotides upstream (from −1 to −15) and downstream (from +3 to +18) from the PTC] from 30 different sequences with reported positive read-through induction and 29 sequences with described negative read-through induction. In sequences with positive read-through induction, TGA was the most represented stop codon (66.6%), followed by TAG (30.0%) and TAA (3.3%) (Fig. 7A). In sequences with absence of read-through induction, there was a clear reduction in the percentage of sequences with TGA stop codon (55.1%) as well as a fourfold increase in the percentage of TAA stop codon (13.8%) (Fig. 7B). Additionally, major differences between sequences with or without read-through induction were that 80% (24/30) of the sequences with read-through induction presented a G or T nucleotide in position −9 versus 52% (15/29) in those with no induction; 73% (22/30) of the sequences with read-through induction presented an A or T nucleotide in position −8 versus 52% in those with no induction; and 50.0% (15/30) of the sequences with read-through induction presented a C nucleotide in position −3 versus 34.5% (10/29) in those showing no induction (Fig. 7A,B). By contrast, the frequency of the C nucleotide in position −2 was more than twofold higher in sequences with no read-though induction (∼41%) than in those showing induction (∼20%) (Fig. 7A,B). Furthermore, the presence of C nucleotide in position +4, which promotes PTC read-through (Dabrowski et al., 2015), was 33% and 21% in sequences with and without induction, respectively (Fig. 7A,B). The estimated consensus sequence for the 30 and 29 sequences with and without induction, respectively, are shown in Fig. 7C and D. Compared to these analyzed sequences, mouse *Pygm* p.R50X sequence presented the TGA stop codon, as well as G at position −9, and C at −3 and −2, but did not present A/G or A at +13 or +14 (Fig. 7E).

## DISCUSSION

In the present work, we report the absence of *Pygm* p.R50X read-through induction in McArdle disease cell cultures using a variety of RTAs. Three different cell culture models were used to evaluate the efficiency of these RTAs in McArdle disease. The first model consisted of HeLa cells transiently transfected with two different GFP-*PYGM* constructs presenting the p.R50X mutation (GFP-*PYGM* p.R50X and *PYGM* Ex1-GFP p.R50X). The rationale for the evaluation of the distinct RTAs in cells transfected with these two constructs was based on their capacity to generate GFP-*PYGM* fusion proteins of different molecular weight, depending on whether p.R50X read-through is induced or not. Additionally, as these two plasmids include mouse *Pygm* cDNA without any intron sequence, their transfection in cells would allow for the analysis of the efficacy of read-through induction with no concomitant NMD interference (Pérez-Ortín et al., 2013). No read-through induction was observed in HeLa cells transfected with either GFP-*PYGM* p.R50X or *PYGM* EX1-GFP p.R50X plasmids when treated with amlexanox, Ataluren or G418. These results are discordant with those obtained by Birch et al. (2013), who reported that G418 induced read-through in Chinese ovary hamster cells transfected with the PYGM EX1-GFP p.R50X plasmid. The study did not proceed with RTC13 as it negatively affected the viability of HeLa cells over time. RTC13 is a non-aminoglycoside low-molecular-mass compound identified in a high-throughput screening of ∼34,000 compounds in the search for read-through activity (Du et al., 2009a). Together with another read-through compound identified in the screening, RTC14, RTC13 has proven to suppress nonsense mutations in ataxia telangiectasia patient cell lines and in myotube cells from the mdx mouse model of Duchenne muscular dystrophy (Kayali et al., 2012; Du et al., 2009b). Remarkably, no major toxicity effects have been reported for RTC13 in any of the mammalian cells tested.

The second cell culture model used to evaluate *Pygm* p.R50X read-through was based on HEK293T cells stably expressing modified versions of the mutated constructs. In this case, we used the *EF1A* (also known as *EEF1A1*) promoter to minimize silencing phenomena, and used flow cytometry to detect small changes in fluorescence in large amounts of cells. Additionally, we have analyzed a wider panel of RTAs (including the aminoglycosides azithromycin, erythromycin, gentamycin and neomycin, as well as the non-aminoglycoside RTC14), but no read-through effect was detected in the experimental conditions used.

The third cellular model evaluated the read-through activity in skeletal muscle cultures derived from the McArdle mouse model. In contrast to what occurs with muscle cultures derived from affected humans, McArdle mouse skeletal muscle cultures do not present GP protein and activity, and accumulate large amounts of glycogen deposits, and therefore represent a suitable *in vitro* model to analyze and evaluate potential treatments for the disease (de Luna et al., 2015). In these cells, NMD of *Pygm* mRNA has been shown to occur, as *Pygm* mRNA levels of myotubes derived from McArdle mice are ∼10% of those from their WT counterparts (de Luna et al., 2015). Thus, this model is useful not only to evaluate the read-through capacity of the different compounds, but also to assess their capacity for inhibiting the NMD mechanism. Although some reports have indicated that a moderate induction of the PTC read-through by using RTAs may promote a stabilization of mutant transcripts and counteract the NMD process (Gonzalez-Hilarion et al., 2012; Floquet et al., 2011; Bellais et al., 2010; Salvatori et al., 2009), we did not find a significant change in the *Pygm* mRNA levels in McArdle mouse skeletal muscle cultures when treated with different doses of amlexanox, Ataluren or G418. Consistent with these findings, we did not detect GP-M protein in any of the conditions tested. Thus, in our experimental conditions, the results obtained in these three cellular models indicate an absence of p.R50X read-through induction capacity of the different RTAs tested – amlexanox, Ataluren, RTC13 and G418. In addition, the aminoglycosides azithromycin, erythromycin, gentamycin, neomycin and RTC14 were unable to induce p.R50X read-through on HEK293T stably transfected cells. Previous research in McArdle patients showed that 10-day administration of gentamicin failed to provoke full-length GP-M production or attenuation of disease manifestations [e.g. no reduction in the levels of serum creatine kinase, a muscle damage biomarker (Schroers et al., 2006)].

The absence of *Pygm* p.R50X read-through induction in our experimental conditions might be multifactorial. Our choice of read-through compounds was meant to reflect different types of compounds, not to cover the entire and constantly expanding spectrum of compounds. Amlexanox, Ataluren, RTC13 and G418 have proven to be effective in read-through induction in several different PTCs both *in vitro* and *in vivo* (Gonzalez-Hilarion et al., 2012; Howard et al., 1996; Heier and DiDonato, 2009; Floquet et al., 2011; Salvatori et al., 2009; Brasell et al., 2019; Yu et al., 2014; Tan et al., 2011; Wilschanski et al., 2011; Du et al., 2008; Sánchez-Alcudia et al., 2012; Schwarz et al., 2015; Martorell et al., 2019). However, it is also important to mention that some criticism has been raised against Ataluren, its mode of action and its ability to induce PTC read-through (Chowdhury et al., 2018; McElroy et al., 2013; Auld et al., 2009; Dranchak et al., 2011). Adequate dosage and incubation time may be factors for each of the tested compounds, but our choice of both relied on past published read-through induction in various *in vitro*/*vivo* conditions (Gonzalez-Hilarion et al., 2012; Lentini et al., 2014; Heier and DiDonato, 2009; Tan et al., 2011; Sánchez-Alcudia et al., 2012; Dranchak et al., 2011; Gómez-Grau et al., 2015). Finally, we evaluated the possibility that the nucleotide context sequence surrounding the p.R50X mutation might have influenced the capacity of the different compounds for read-through induction. It has been reported that the stop codon sequence influences read-through induction; in this regard TGA>TAG>TAA is the order in which the different stop codons present higher read-through potential (Dabrowski et al., 2015). Additionally, experimental studies in a number of organisms have shown that both downstream and upstream sequence context plays an essential role in determining the read-through potential of stop codons. In both bacteria and eukaryotes, the base immediately following the stop codon exerts the strongest influence on read-through efficiency. For example, the level of basal TGA-C read-through in mammalian cells (3-4%) was shown to be three to six times higher than for the remaining TGA-tetranucleotides (Dabrowski et al., 2015). In yeast (*Saccharomyces cerevisiae*), at least six nucleotides after the stop codon are a key determinant of read-through efficiency (Namy et al., 2001). Regarding the PTC upstream sequence context, in experiments performed with mouse fibroblasts (NIH3T3) and human HEK293T cell lines, the highest read-through occurrence was observed when the −1 position (the first nucleotide of the stop codon is assigned as +1) was occupied by adenine or generally purine; in these models, uracil at the −1 position was always associated with the lowest read-through level (Dabrowski et al., 2015). In this respect, our analysis of 30 PTCs and their associated context sequences that presented positive read-through induction, in comparison to 29 different sequences with negative read-through induction, suggest a potential role of nucleotide positions −9, −8, −3, −2, +13 and +14. The mouse *Pygm* p.R50X mutation presents TGA as a stop codon, G nucleotide at positions −1 and −9, and C nucleotide at −3, which potentially generate a good context for read-through induction, counteracted by the presence of C at −2 and its absence at +4. Similarly, human *PYGM* p.R50X context sequence presents homology of ∼85% with the mouse sequence and the potential read-through hot spots are conserved among both species.

Overall, amlexanox, Ataluren, RTC13 and G418 failed to induce p.R50X read-through in three McArdle disease cell culture models containing the mouse *Pygm* sequence. It is likely that these would also fail to induce read-through in the human sequence based on the complete conservation of the read-through hotspots. However, further studies confirming these results in the human *PYGM* p.R50X sequence, as well as using newer RTAs such as NB74, NB84, NB124, GJ071, GJ072, BZ6, BZ16 and Clitocine among others, could provide a better understanding of the necessary molecular requirements and spatial allowance in the ribosome, as it has been described that as little as 2% of normal GP-M activity might be enough to ameliorate the disease phenotype in patients (Vissing et al., 2009).

## MATERIALS AND METHODS

### Plasmid constructs

Four constructs encoding the WT and mutant (p.R50X) GP-M and enhanced green fluorescent protein (GFP) fusion proteins were a kind gift from Dr Glenn E. Morris (Birch et al., 2013). The *Pygm* sequence used in these constructs corresponded to the mouse sequence (NCBI Accession BC012961) (Birch et al., 2013), as mouse and human GP-M amino acid sequences have high identity (97%). Two of these consisted of N-terminal GFP fused to WT and p.R50X GP-M full-length sequences inserted into a pCI-neo vector, while the other two consisted of C-terminal fusions of GFP to the N-terminal 50 amino acids of WT or 49 amino acids of p.R50X GP-M protein (Fig. 1A,B). The pCI-neo vector provides the cytomegalovirus immediate early promoter (pCMV). In order to check the sequence of the constructs and the presence/absence of the p.R50X mutations, the four plasmids were sequenced using the primer comprising the beginning of the mouse *Pygm* coding sequence (5′-ATGTCCAGGCCTCTTTCAGACCAG-3′), T7 promoter primer (5′-TAATACGACTCACTATAGG-3′) and T3 promoter primer (5′-ATTAACCCTCACTAAAGGG-3′) surrounding the pCI-neo multicloning site. The GFP coding sequence from the four constructs was disrupted by the *XhoI* restriction site used in the cloning process and, as a consequence, the last 20 amino acids from the GFP protein were missing (from Ala_219_ to Arg_238_) (Fig. 1A,B). In addition, the WT and p.R50X GFP-*PYGM* constructs included 18 amino acids that resulted from the translation of the 5′UTR *Pygm* sequence included in the construct because of *XhoI* and *MluI* restriction site usage during the cloning process (Fig. 1A). Finally, the WT and p.R50X *PYGM*-GFP constructs included 32 amino acids between the disrupted GFP protein and the first stop codon of the pCI-neo vector (Fig. 1B). For the stable expression of the constructs, the WT and p.R50X *PYGM*-GFP sequences were subcloned into the pCMV/blasticidin S (Bsd) vector in which the expression of the WT and p.R50X *PYGM*-GFP (C-terminal fusions) was driven by the *EF1A* promoter and contained the Bsd resistance gene as a selectable marker (Fig. 1C).

### Transient transfections

HeLa cells were obtained from American Type Culture Collection (ATCC; Manassas, VA, USA) and tested for contamination. These cells were grown on six-well plates with Dulbecco's modified Eagle medium (DMEM) high glucose (Biowest, Nuaillé, France) plus 10% fetal bovine serum (FBS; Gibco™, Thermo Fisher Scientific, Waltham, USA), 100× 1% non-essential amino acids, 200 mM L-glutamine, 100 mM sodium pyruvate (all Biowest) and 100× penicillin/streptomycin (Pen-Strep solution, Biological Industries, Kibbutz Beit HaEmek, Israel), and incubated at 37°C, 5% CO_2_ and 100% relative humidity. When the cells reached 70% confluence, different amounts of each construct (0, 0.5, 1 1.5, 2 and 2.5 µg) were added to different wells following the Lipofectamine 3000 Transfection Reagent (Invitrogen, Carlsbad, CA, USA) protocol.

### Generation of stable cell clones expressing the constructs

HEK293T cells were obtained from ATCC and tested for contamination. These cells were cultured in DMEM high glucose supplemented with 10% FBS, 200 mM 1% L-glutamine (all from Biowest) and 100× penicillin/streptomycin (Capricorn Scientific, Ebsdorfergrund, Germany) and incubated at 37°C, 5% CO_2_ and 100% relative humidity. Cells were seeded in a 24-well plate so that confluence at the time of transfection, 24 h after seeding, was 70% (0.5-10×10^5^ cells/well). Both plasmids (pC-*PYGM* Ex1-GFP WT and p.R50X) were transfected with Lipofectamine 3000 Transfection Reagent (Invitrogen), following the manufacturer's instructions. Non-transfected cells were used as a control. Forty-eight hours after transfection, cells were treated with Bsd (Gibco™) at 15 µg/ml, a concentration that was found to be optimal for selection in preliminary experiments (data not shown). Bsd was added to the cultures on the day of seeding and replaced every 3-4 days with the cell passages. Bsd-resistant cells expressing the WT construct were analyzed by flow cytometry after 7 days of selection to determine GFP expression. In order to obtain more homogeneity, at this point, single cells were isolated using a FACSAria™ I cell sorter (BD Biosciences, Franklin Lakes, NJ, USA) and individual clones were expanded in culture in the presence of Bsd. In the case of cells carrying the WT construct, the clones were generated from the GFP^+^ cell population. Non-transfected and transfected untreated cells were used as controls.

### Skeletal muscle cultures

WT and skeletal muscle cultures derived from the McArdle mouse model were obtained as previously reported (de Luna et al., 2015). Myoblasts were grown in Ham F10 medium (Biowest) supplemented with 20% FBS (Gibco™), 1% glutamine (Biowest), Pen-Strep solution (Biological Industries) and 20 ng/ml basic fibroblast growth factor (PeproTech, London, UK) on 10-cm dishes until 90% confluence. Then, cells were transferred to six-well plates and grown in DMEM high glucose (Biowest) supplemented with 2% horse serum (Gibco™), 1% glutamine (Biowest) and Pen-Strep solution (Biological Industries) for myotube differentiation.

### Treatment with RTAs

A variety of RTAs were tested in the different cellular models using a wide range of concentrations (Table 1). Treatment was initiated 8 h after transfection in HeLa cells, while in the skeletal muscle cultures it was started after 48 h in differentiation medium. In both cases, the treatment was maintained for 72 h [based on previous reports (Lentini et al., 2014; Du et al., 2013; Nakamura et al., 2012; Howard et al., 1996; Heier and DiDonato, 2009; Friesen et al., 2018; Floquet et al., 2011)] and the medium was changed every day. In the case of the HEK293T stable cell clones, treatment was initiated 24 h after seeding and maintained for 48 h.

### mRNA analyses

Total RNA was obtained from cultured cells following the manufacturer’s instructions for TRIzol (Invitrogen). RNA was treated with DNase I, amplification grade (Invitrogen) to eliminate any traces of DNA. cDNA was synthesized from RNA using a high-capacity cDNA archive kit (Applied Biosystems, Foster City, CA, USA), which uses random primers. We used real-time polymerase chain reaction (PCR), with TaqMan fluorogenic probes in a 7900 Real-Time PCR System (Applied Biosystems) to assess *Pygm* RNA levels (Mm00478582_m1). Results were normalized to peptidylprolyl isomerase A (cyclophilin A, *Ppia*) mRNA levels (probe Mm02342430_g1) and quantified using 7900 SDS v2.4.1 software (Applied Biosystems).

### Western blot analysis

Protein extraction from transfected cells and skeletal muscle cultures was performed by adding extraction buffer (20 mM Tris-HCl pH 7.5, 150 mM NaCl and 1% Triton X-100) to each well. Then, cells were sonicated for 5 s (one cycle 100% amplitude) with a Labsonic® M homogenizer (Sartorius Biotech, Göttingen, Germany). Equal amounts of extracted proteins (30 μg) were resolved on Criterion™ TGX™ 4-15% Precast Midi gels (Bio-Rad, Hercules, CA, USA) at 150 V for 90 min and blotted to a polyvinylidene difluoride (PVDF) membrane (Immun-Blot® PVDF membrane, Bio-Rad) using a Trans-Blot® SD Semi-Dry Transfer Cell (Bio-Rad) at 20 V for 50 min. Membranes were incubated in anti-GFP (A6455, Thermo Fisher Scientific; 1:2000) or anti-PYGM (19716-1-AP, Proteintech™, Rosemont, IL, USA; 1:2000) primary antibodies overnight at 4°C and in horseradish peroxidase (HRP)-conjugated goat anti-rabbit (Jackson Laboratories, Baltimore Pike, PA, USA) secondary antibody for 3 h at room temperature. Ponceau S staining (Sigma-Aldrich, St Louis, MO, USA) was used as a loading control for all the membranes. Membranes were developed with Immobilon Western Chemiluminescent HRP Substrate (Merck-Millipore, Burlington, MA, USA) and images were obtained with an Odyssey^®^ Fc Imaging System (LI-COR Biosciences, Lincoln, Nebraska, USA) and quantified with ImageJ, version 1.45 (National Institutes of Health, Bethesda, MD, USA; http://imagej.nih.gov/ij).

### Flow-cytometric analysis of stable HEK293T cell clones treated with RTA

Transfected and non-transfected cells were seeded in a flat-bottom 96-well plate (8000 cells/well) in the presence of Bsd. After 24 h, treatment was started using the RTAs described in Table 1. Cells were analyzed by flow cytometry 48 h after treatment initiation. Treated cells were labeled with the viability marker 7-aminoactinomycin D (7-AAD). Flow cytometry was performed with BD LSRFortessa^TM^ (BD Biosciences) and results were analyzed with FCS Express 4 Software (De Novo Software, Pasadena, CA, USA). Experiments were performed in duplicate for all conditions.

### Literature search

In order to establish a consensus sequence for positive and negative read-through induction, a number of gene sequences with PTCs and treated with RTAs were analyzed and separated according to positive or negative outcome (Du et al., 2013, 2009b, 2006; Kayali et al., 2012; Howard et al., 1996; Floquet et al., 2011; Tan et al., 2011; Sánchez-Alcudia et al., 2012; Schwarz et al., 2015; Martorell et al., 2019; Dranchak et al., 2011; Bukowy-Bieryllo et al., 2016; Linde et al., 2007; Harada et al., 2018; Lincoln et al., 2018). All sequences corresponded to human gene sequences with the exception of the mouse *Cftr* G542X (Du et al., 2009b, 2006) and *Dmd* Q995X (Kayali et al., 2012) gene sequences. For each gene, the surrounding PTC sequence including 15 nucleotides upstream and downstream from the reported PTC (−15 to +18; being +1 the first nucleotide of the PTC) was obtained using the consensus coding sequence (CCDS) project database (https://www.ncbi.nlm.nih.gov/CCDS/). Sequences were aligned with Multiple Alignment using Fast Fourier Transform (MAFFT) (https://www.ebi.ac.uk/Tools/msa/mafft/; output ClustalW). Aligned sequences were reformatted using MView (https://www.ebi.ac.uk/Tools/msa/mview/; parameters: width 80, coloring identity, color map D2 and group map none). Additionally, manual annotation was performed; nucleotides that were represented ≥35% and ≥30% among the aligned sequences were marked in blue and purple, respectively. Finally, to obtain the consensus sequences, the combination of the WebLogo program (https://weblogo.berkeley.edu/logo.cgi) along with manual annotation was used; thus, for each position the consensus nucleotide was defined as a single nucleotide with a frequency ≥50%, or when the combination of two nucleotides generates a frequency ≥70% (only when each nucleotide had a frequency ≥30%).

## Supplementary Material

Supplementary information
