# Supplementary material for: Absence of p.R50X Pygm read-through in McArdle disease cellular models
Source: Dis Model Mech. 2020 Jan 13;13(1):dmm043281. doi: 10.1242/dmm.043281 (PMC6994938; doi:10.1242/dmm.043281)
Supplement: Supplementary information [file dmm-13-043281-s1.pdf]

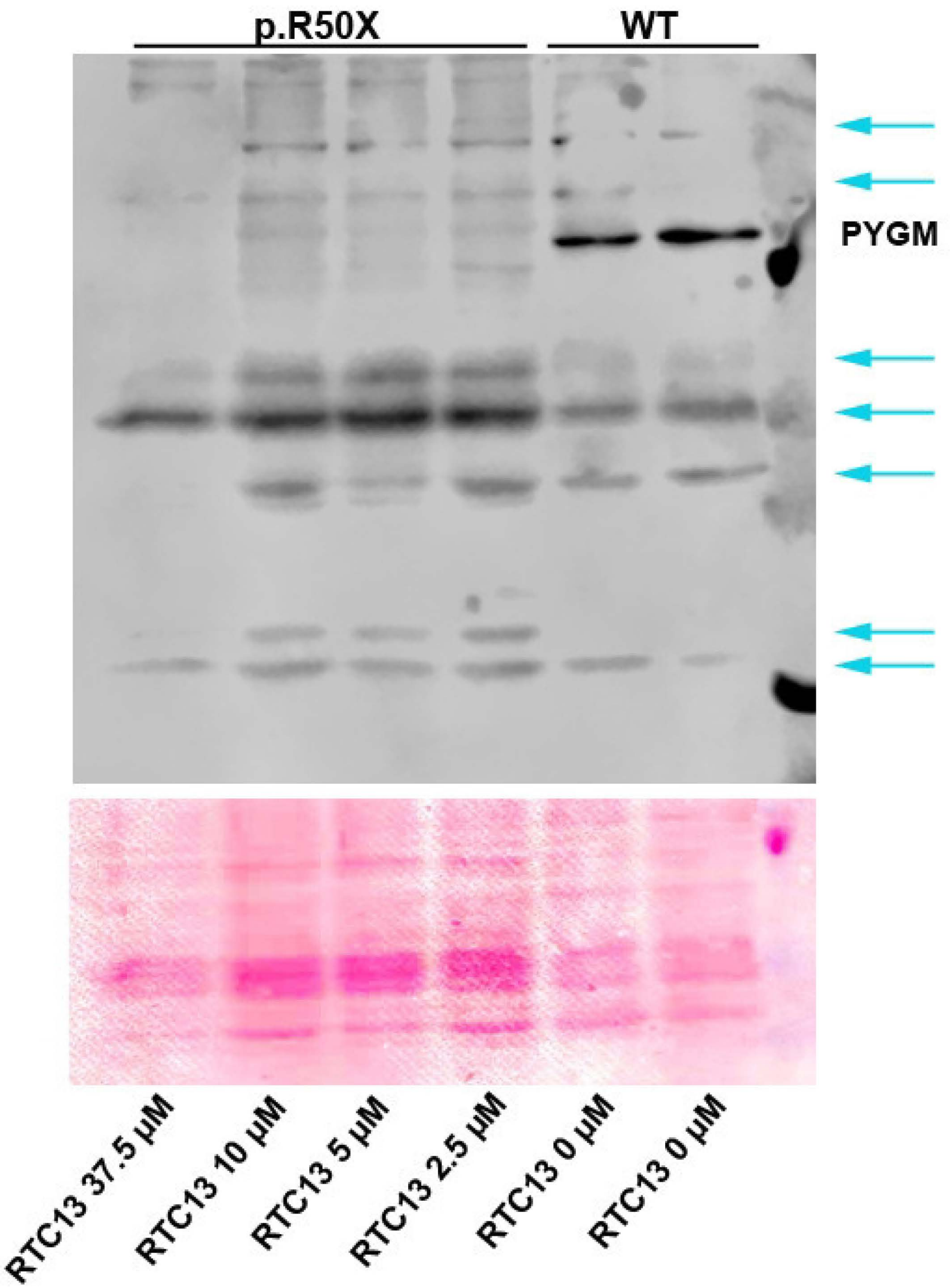

Figure S1. GP-M protein levels in WT and McArdle mouse myotubes after 72 hours treatment with different concentrations of RTC13. Dark arrows mark the specific GP-M protein band (94 kDa), while blue arrows mark unspecific protein bands.

Disease Models & Mechanisms • Supplementary information
